# Evaluation of Food Supply Security for Grain and Key Agricultural Products in Gansu Province Under the Comprehensive Food Security Perspective

**DOI:** 10.3390/foods15152687

**Published:** 2026-07-30

**Authors:** Yifei Yang, Bo Dong, Wenhui Liang, Dandan Du, Zhenhua Zhao, Li Jia, Tao Huang

**Affiliations:** 1 College of Finance and Economics, Gansu Agricultural University, Lanzhou 730070, China; 2 Key Laboratory of Efficient Utilization of Water in Dry Farming of Gansu Province, Dryland Agriculture Institute, Gansu Academy of Agricultural Sciences, Lanzhou 730070, China; 3 Key Laboratory of Low-Carbon Green Agriculture in Northwestern China, Ministry of Agriculture and Rural Affairs, Lanzhou 730070, China; 4 Dingxi Agricultural Technology Extension Station, Dingxi 743000, China

**Keywords:** comprehensive food perspective, grain and key agricultural products, supply security assessment, entropy-weighted TOPSIS model

## Abstract

To evaluate the level of food and key agricultural product supply security in Gansu Province, this study constructed a comprehensive evaluation index system based on five dimensions—supply quantity, quality, structure, economic security, and distribution security—and applied the entropy-weighted TOPSIS model to assess the 14 prefectures and cities in Gansu Province from 2018 to 2023. The results show that: (1) Under the “comprehensive food security” framework, the level of food security in Gansu Province has generally improved steadily, with the indices for all prefectures and cities showing an overall positive trend. (2) Geographically, there has been an overall improvement, but growth rates have varied across regions: the central and eastern parts of the Hexi Corridor and the Longdong region have seen strong growth, while growth in parts of the Longzhong and Longnan regions has been relatively slow. (3) From various perspectives, the quality and safety of supply are at the highest level; the economic security of supply exerts a strong driving force; the quantity security of supply is improving amid fluctuations, working together with distribution security to strengthen the system’s foundation; and the structural security of supply is being adjusted and optimized amid fluctuations. Based on this, while consolidating advantages in quality and growth drivers, efforts must be focused on addressing regional development imbalances to promote balanced development across the entire region.

## 1. Introduction

Ensuring the supply of grain and agricultural products is the most fundamental function of agriculture [1]; food security is a top priority in state governance and serves as the “stabilizer” and “ballast” for ensuring national security [2]. China’s grain production has repeatedly set new records; in 2021, per capita grain availability reached 483 kg, far exceeding the international food security threshold, achieving basic self-sufficiency in grains and absolute security in staple food supplies [3]. Currently, China’s agriculture is undergoing a systemic transition from a traditional factor-driven model to one driven by technology, institutional reforms, and green values [4]. As we enter a new era, the institutional framework and working mechanisms for the development of agriculture, rural areas, and farmers must focus on key areas such as national food security and the improvement of farmers’ livelihoods [5], and consolidate the foundation of food security at a high level through multiple avenues, including ensuring overall supply, improving quality, optimizing structure, promoting ecological sustainability, and diversifying supply sources [6]. Optimizing the food structure while ensuring absolute food security represents a strategic shift toward a broader concept of food security.

Early research has clarified the relationship between food security and food, nutrition, and ecological security [7]. Cheng Guoqiang [8] further elaborated on its policy implications: it lies in expanding the concept of food security to encompass food safety, emphasizing the synergy among a comprehensive approach to resources, agriculture, and markets; Li Yihong and Huang Ying [9] point out that the “comprehensive food perspective” places higher demands on resource utilization, crop structure, the supply system, and coordinated development. Putting the comprehensive food perspective into practice, advancing the transformation of arable land use, and building a diversified supply system are key pillars [10]; among these, the phenomenon of farmland being diverted from grain production is a concentrated manifestation of the contradictions arising during this transformation. In response, the academic community has used methods such as the Maxent model to quantify the risks of non-food crop cultivation and its impact on yields [11], while Fan Shenggen et al. [12] emphasize the need to develop diverse food sources from a holistic perspective of national land resources to address structural shortages; Liang Yajia and Chen Kunqiu [13], on the other hand, have highlighted the dual risks of nutritional structural imbalances and spatial mismatches from the perspective of nutritional supply and demand, advocating for a goal-oriented shift in nutrition and health policy from “production-driven demand” to “demand-driven production”. International research on the potential of duckweed as a protein substitute in diversified food systems [14] suggests that improved water resource management can increase food self-sufficiency in sub-Saharan Africa [15]; ensuring the continued supply and availability of food during prolonged disruptions remains a key challenge facing agri-food supply chains [16].

How to scientifically and quantitatively evaluate food security within the framework of a “broad food perspective” has become a key research focus. At the evaluation system level, Wang Huogen et al. [17] developed a multidimensional indicator system and found that production security made a significant contribution, while consumption and distribution were the weak links; another study comprehensively considered regional food supply dynamics and differences in dietary habits to construct a provincial-level food security evaluation system for China [18]. In terms of evaluation methods, Kousar et al. used two-factor principal component analysis to construct a food safety index [19]; Pu Mianzhe et al. [20] explored the predictive applications and prospects of machine learning, and Yang Chuan et al. [21] identified spatial clustering and club convergence phenomena in food security levels. Yang et al. [22], using a coupled coordination model and the Moran’s I index, found that the level of coordination between agricultural production and trade in China is higher in the east than in the west and generally low overall. The key to ensuring supply security lies in enhancing the resilience of the agricultural industrial chain and promoting systemic transformation. Zhang Yumei and Long Wenjin [23] systematically analyzed the resilience challenges facing China’s industrial chain in areas such as technology, resources, and markets; Yan Tingwu and Huang Yazi [24], using edible fungi as an example, outlined a path toward a diversified, efficient, and sustainable agri-food system. Liu Changquan et al. [25] focused on the mismatch between grain and forage production and emphasized the need to ensure a minimum level of self-sufficiency in staple foods; Zhang Liming’s [26] discussion of the “grain-to-forage conversion” initiative in Gansu represents a localized response to this issue. Enhancing resilience and driving transformation are inseparable from technology-driven development. The “new-quality productive forces in agriculture” proposed by Huang Jikun [27] centers on a significant increase in total factor productivity, which relies on innovations in biotechnology, digital technology, and other fields. Wang Xiaojun and Mao Shiping [28], meanwhile, have identified key scientific and technological fields and pointed out that Gansu should focus on the technological self-reliance of the seed industry and protected agriculture, as well as the timeliness of financial tools such as agricultural insurance [29]. Resilience ultimately takes root at the micro level of the household. Studies such as those by Odunitan-Wayas et al., which examine dietary differences among older adults under varying food security conditions [30], and Annet Adong’s analysis of household food insecurity and women’s dietary diversity resilience using high-frequency data [31], provide a micro-level perspective for understanding end-user vulnerability and resilience. At the same time, market price signals are crucial for adjusting production [32]. Studies have shown that fluctuations in grain and agricultural product prices have lagged effects [33]; therefore, when assessing supply security in Gansu, attention must be paid to the transmission effects of price fluctuations on farmers’ production decisions.

However, existing studies suffer from issues such as a narrow selection of evaluation indicators and highly subjective weightings, making it difficult to accurately reflect the actual level of regional supply security. This paper employs the entropy-weighted TOPSIS integrated model: the entropy-weighting method is used to objectively determine weights; a weighted matrix is constructed to calculate proximity (the closer to 1, the better the performance); and information entropy weighting is applied to avoid subjective bias and enhance decision-making reliability. This model demonstrates good applicability in scenarios involving multiple indicators and diverse samples [34]. This article constructs an evaluation system for the supply security of grain and key agricultural products based on five dimensions—quantity, quality, structure, economy, and distribution—and conducts an empirical study on grain and key agricultural products in Gansu Province. Building on existing domestic research, this paper conducts a subregional analysis based on international multidimensional food security assessment paradigms and establishes an evaluation system using the nutritional indicators of locally distinctive and high-quality agricultural products to enhance the international comparability of the research.

## 2. Analysis of the Current Status of Grain and Key Agricultural Products in Gansu Province

### 2.1. Stable Grain Production and Supply Assurance in Gansu Province

As shown in Table 1, between 2018 and 2023, the province’s population declined from 25.1508 million to 24.6548 million, while the number of rural workers fell from 7.89 million to 6.81 million, a decrease of 1.08 million, far exceeding the decline in the total population. As a result, agricultural production methods had to be upgraded, and mechanization increased continuously, enabling total grain output to keep growing despite the shrinking labor force.

**Figure 1:**
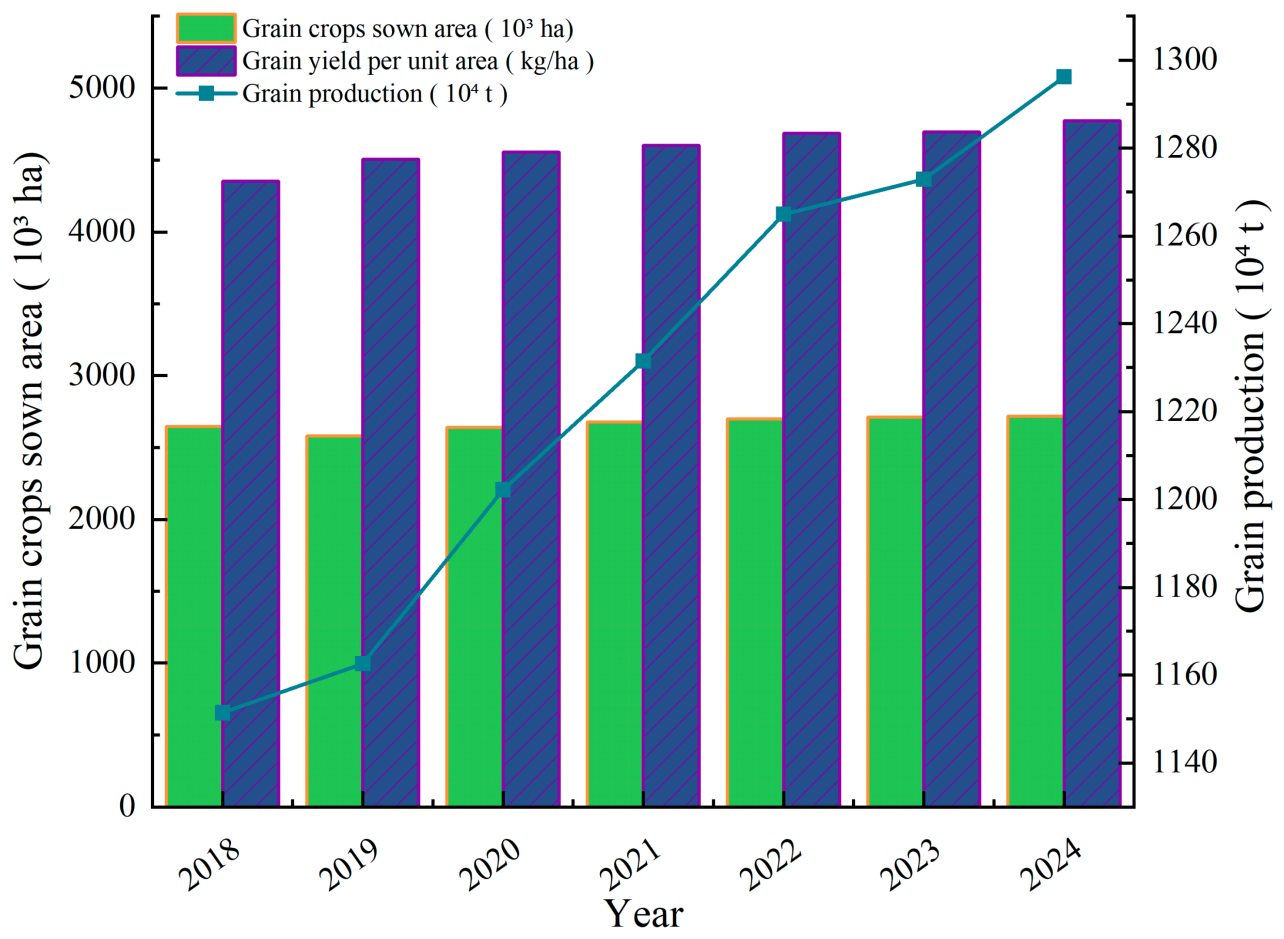
Changes in Grain Production and Cultivated Area in Gansu Province.

As shown in the trends in grain production and cultivated area (Figure 1), the area planted with grain crops showed a steady upward trend between 2018 and 2023, gradually increasing from 2645.26 thousand hectares in 2018 to 2710.93 thousand hectares in 2023, representing a cumulative increase of approximately 2.48%. At the same time, the upward slope of the grain production curve has steepened significantly since 2020, indicating that grain production has entered a phase of accelerated growth; in 2023, grain yield per unit area reached 4695.44 kg. Gansu’s local grain reserves total 2.939 million metric tons, representing 150.7% of the national target level. This capacity is sufficient to cover 7.5 months of the province’s consumption. Of this total, 1.325 million metric tons are held at the provincial level and 1.614 million metric tons at the municipal and county levels. The primary grain is wheat, with small quantities of corn and barley. Within the grain reserve quota, 116,000 metric tons of emergency finished flour and rice reserves have been established, sufficient to cover more than 15 days of consumption in provincial and municipal capitals and 13 days of consumption across the entire province. The province’s per capita grain reserves rank among the highest in the country.

### 2.2. Supply Capacity of Gansu’s Specialty Agricultural Products

In terms of industrial structure (Table 2), vegetable production ranks first. Among the province’s specialty industries, vegetable production ranks first, reaching 18.2265 million tons in 2023; followed by orchard fruits, with a production volume of 6.1640 million tons; in terms of livestock products, mutton production was 409,000 tons, and beef production was 297,600 tons; among specialty crops, tuber production was 2.2210 million tons, and medicinal herb production was 1.4890 million tons. In terms of output growth, mutton and fruit production have grown rapidly. Growth rates vary across different sectors. Between 2018 and 2023, mutton and orchard fruit production saw average annual growth rates exceeding 10%, indicating relatively rapid growth; beef, vegetables, and medicinal herbs maintained steady growth of around 7%; and tuber production remained stable.

### 2.3. Grain and Key Agricultural Product Consumption in Gansu Province

**Figure 2:**
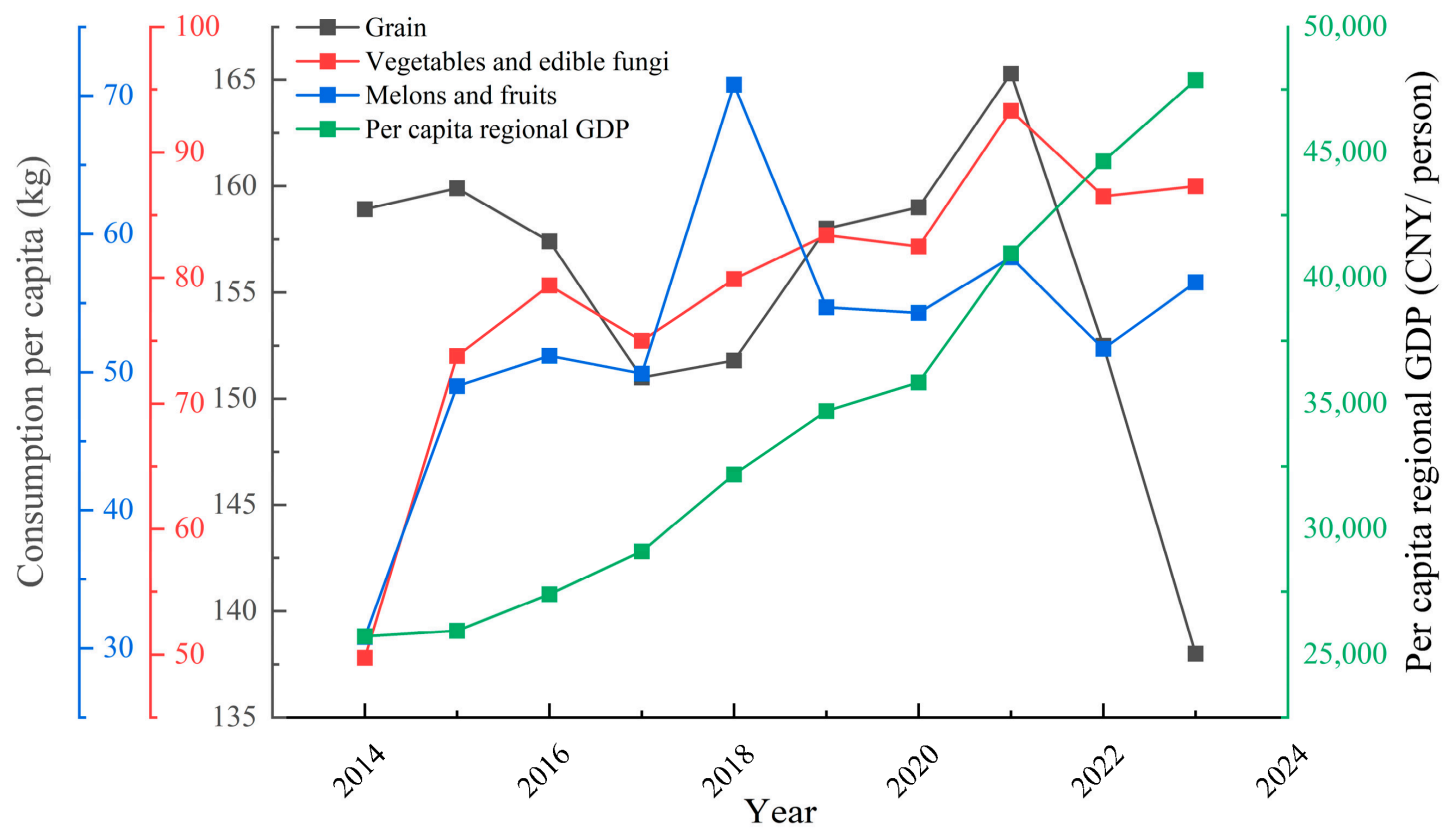
Per capita GDP and per capita food consumption in Gansu Province, 2014–2023.

The food consumption structure follows Bennett’s Law. With the economic and social development of Gansu Province and the improvement in residents’ living standards, consumption of starchy staples such as rice and wheat flour has gradually decreased, while consumption of vegetables, and fruits has increased significantly, reflecting Bennett’s Law in the food consumption structure. As shown in Figure 2, Per capita grain consumption fell from 158.9 kg in 2014 to 138 kg in 2023, representing an average annual decline of 1.5%, while its share of total food consumption dropped from 57.2% to 39.7%; Meanwhile, per capita consumption of vegetables, and fruits increased annually by 6.5%, and 7%, respectively, with their shares of total food consumption rising from 17.9%, and 11.1% to 25.1%, and 16.3%, respectively.

## 3. Materials and Methods

### 3.1. Data Sources

Based on data release schedules and the availability of indicators, this analysis examines the level of food security for grain and key agricultural products in Gansu Province from 2018 to 2023, as part of the “Comprehensive Food Security” framework. The relevant raw data are sourced from the *Gansu Statistical Yearbook* and the *Gansu Rural Yearbook*, and the statistical bulletins on national economic and social development issued by various prefectures and cities. For a small number of indicators, such as the contribution rate of agricultural scientific and technological progress, the length of high-speed freight rail lines, and per capita fish consumption, there were minor data gaps in certain years for some prefectures and cities. These missing data accounted for 3.3% of the total valid sample and were imputed using linear interpolation. At the same time, the robustness tests presented later confirm that the linear interpolation process did not cause any structural disturbances to the relative rankings or spatial patterns of the evaluation results, and the data quality meets the requirements for the empirical analysis in this study.

### 3.2. Model Construction

The security of grain and key agricultural product supplies involves a multidimensional evaluation, which is assessed using the entropy-weighted TOPSIS method. This method objectively assigns weights to determine the importance of indicators using the entropy-weighting approach, calculates the relative proximity of each prefecture and city to the optimal and worst-case supply scenarios, and establishes a ranking of the evaluated entities. It offers advantages such as intuitive geometric representation, minimal information loss, and operational flexibility [35].

#### Research Methods

(1) Standardization Processing

To address differing measurement scales among indicators, the range method is applied to standardize all measurement indicators in the Gansu Province food supply security evaluation system and the broad food concept ${X\prime }_{ij}$ represents the raw value of the *j*th measurement indicator for the *i*th prefecture; ${X\prime }_{ij}$ denotes the standardized value of the *j*th measurement indicator for the *i*th prefecture; $max(x_{j})$ and $\min\left(x_{j}\right)$ denote the maximum and minimum values of ${X\prime }_{ij}$, respectively.

(1) $$X_{ij}^{\prime }=\frac{x_{ij}-\min\left(x_{j}\right)}{\max\left(x_{j}\right)-\min\left(x_{j}\right)}(Positive\;indicators)$$

(2) $$X_{ij}^{\prime }=\frac{\max\left(x_{j}\right)-x_{ij}}{\max\left(x_{j}\right)-\min\left(x_{j}\right)}(Negative\;Indicators)$$

(2) Calculate the weight $P_{ij}$ for the *i*th year under the *j*th indicator,
  (3) $$P_{ij}=\frac{X_{ij}^{\prime }}{\sum_{i=1}^{Ν}X_{ij}^{\prime }}$$

(3) Calculate the entropy value $e_{j}$ of the *j*th indicator
  (4) $$e_{j}=-k\sum_{i=1}^{N}P_{ij}\ln(P_{ij}),Where\;k=\frac{1}{\ln(N)},and\;k>0,such\;that\;e_{j}\geq 0.(Sample,\;N=80)$$

(4) Calculate the redundancy of the information entropy for each measure indicator in the evaluation indicator system
  (5) $$d_{j}=1-e_{j}$$

(5) Calculate the weight $\omega_{j}$ for the *j*th indicator
  (6) $$\omega_{j}=\frac{d_{j}}{\sum_{j=1}^{m}d_{j}}(m=20)$$

(6) Calculate the weighted matrix of measurement indicators
  (7) $$Z_{ij}=x\prime_{ij}\times \omega_{j}$$

(7) Calculate the Euclidean distance based on the weight matrix

Where the positive ideal solution distance ($D_{i}^{+}$) indicates the gap between each prefecture-level city’s indicator system and the theoretically optimal safety state; a smaller value indicates a supply safety level closer to the ideal state. The negative ideal solution distance ($D_{i}^{-}$) reflects the deviation of each prefecture-level city’s indicator system from the theoretically minimum safety threshold; a larger value indicates greater distance from the risk-critical state.

(8) $$D_{i}^{+}=\sqrt{\sum (Z_{ij}-Z_{j}^{*+}{)}^{2}}$$

(9) $$D_{i}^{-}=\sqrt{\sum (Z_{ij}-Z_{j}^{*-}{)}^{2}}$$

(8) Calculate the relative proximity of each prefecture-level city to both the optimal and worst-case supply states

$C_{i}$ represents the degree to which the grain and key agricultural product supply security level of the *i*th evaluation subject approaches the optimal level, commonly referred to as the proximity level. Its value ranges from [0, 1]. A larger $C_{i}$ indicates a higher level of grain and key agricultural product supply security.

(10) $$C_{i}=\frac{D_{i}^{-}}{D_{i}^{+}+D_{i}^{-}}$$

### 3.3. Establish an Evaluation Indicator System

Based on a systemic perspective of the “big food” concept, this study establishes an evaluation system for the supply security of grain and key agricultural products in Gansu Province. Supply security is subject to the complex interplay of multiple factors, including resources, the environment, technology, the economy, and policy; these factors are interrelated and interact synergistically, forming a dynamic and organic whole. Drawing on the classic framework of the Global Food Security Index (GFSI) and incorporating the High Level Panel of Experts (HLPE) (2020) report’s concept of integrating sustainability and agency into the dimensions of food security, while combining theoretical analyses of food security, and grounded in Gansu’s agricultural resource endowments and development needs, and drawing on the research by Yang Yueyuan et al. (2025) [35] and Zhang Jiawei et al. (2026) [36], we ultimately constructed a comprehensive evaluation index system for the supply security of food and key agricultural products in Gansu, encompassing multiple dimensions such as quantitative security, quality security, structural security, economic security, and distribution security (Table 3).

Specifically, first, quantitative security is a prerequisite for the security of the supply of grain and key agricultural products. A country’s endowment of agricultural resources determines the level of input of production factors into agricultural production. According to the theory of factor endowment, comprehensive agricultural production capacity is determined by the quantity and allocation efficiency of production factors such as land, labor, and capital; quantitative security focuses on the degree to which these production factors are secured. Per capita arable land area is used to measure crop production and land carrying capacity; the educational attainment of the rural workforce and agricultural laborers reflects the quantity and quality of labor input; and total agricultural machinery power and the contribution rate of agricultural scientific and technological progress measure the level of capital investment and the capacity for scientific and technological support. Second, food safety and quality are inherent requirements of the “big food” perspective. As agriculture undergoes modernization, traditional production methods that rely excessively on chemical inputs to increase yields are no longer sustainable. Agricultural non-point source pollution caused by chemical fertilizers and pesticides directly affects the safety of agricultural products and indirectly reflects the environmental impact and ecological sustainability of agricultural production. Fertilizer application rates and pesticide usage are selected as negative indicators to measure the degree of environmental sustainability in agricultural production; the lower the values, the lower the level of non-point source pollution, and the lower the risks to agricultural product quality and safety as well as the pressure on the ecological environment. Third, the “comprehensive food perspective” calls for a diversified food supply. The structural security dimension focuses on the allocation relationship between food crops, cash crops, and oilseeds within the crop sector, aiming to measure the degree of diversification of agricultural resources at the level of arable land use. Due to limitations in municipal and prefectural-level statistical data, information on the structural ratios between animal husbandry and crop farming, as well as data on the structure of the food processing industry, is not yet available. Therefore, these aspects are currently represented solely by the grain-to-cash crop ratio and the grain-to-oilseed ratio. Both are positive indicators; the grain-to-cash crop ratio serves as the core reference indicator for crop structure adjustment, reflecting the balanced relationship between grain crops and cash crops in the agricultural structure, while the grain-to-oilseed ratio reflects the balanced production relationship between grain crops and oilseed crops. Balancing the grain-to-cash crop ratio and the grain-to-oilseed ratio is a measure to avoid a monotonous crop structure and enhance the resilience of the agricultural system. Fourth, economic security is a key indicator for measuring agricultural output and the nutritional balance of residents’ diets. This study focuses on evaluating the physical capacity of the supply side, covering both total output and per capita nutritional supply. At the output level, the total value of agricultural, forestry, animal husbandry, and fishery production is selected to reflect the overall scale of the agricultural economy; at the per capita level, “the FAO defines availability as an adequate supply of food, including dietary energy and nutrient requirements met through domestic production, imports, and reserves”. Including animal-based foods, fruits, and vegetables in the supply security assessment reflects the need for a nutritional transition from merely “having enough to eat” to “eating well” and is also consistent with the concepts behind indicators such as protein quality in the Global Food Security Index (GFSI). Therefore, in addition to selecting per capita grain consumption as the core indicator, and to implement the requirements of the “comprehensive food security” approach, the analysis includes per capita consumption of cotton, oilseeds, and sugar; per capita consumption of dairy products; per capita consumption of eggs and poultry; per capita consumption of meat; per capita consumption of aquatic products; and per capita consumption of fruits, reflecting the contributions of Gansu Province’s distinctive agricultural sectors, such as cattle and sheep farming and highland summer vegetables; At the same time, average protein supply was selected as a comprehensive indicator to measure nutritional quality and dietary health levels. Fifth, distribution security serves as the link between production and consumption. In the event of a public health emergency or extreme weather, a smooth logistics system is essential for ensuring an uninterrupted food supply. Distribution security focuses on modern food distribution capabilities, using two positive indicators: the density of transportation routes and the mileage of express freight highways. The density of transportation routes reflects the breadth of access and coverage of a region’s road network, while the mileage of expressway freight routes indicates the capacity to ensure timely, long-distance, and highly efficient grain transportation, a fundamental prerequisite for bridging the “last mile” of supply.

It should be noted that, first, this framework focuses on evaluating the physical capacity of regional supply-side security and does not include indicators of affordability or economic accessibility. Economic security focuses on the scale of agricultural output and the stability of producers’ incomes, which fall under the economic foundation of the supply side; affordability, on the other hand, involves the ratio of food prices to household income and relies on data from household consumption expenditure surveys. Gansu Province’s current system of publicly available statistics at the prefecture and city levels does not yet provide such microdata. Second, regarding dietary diversity, although the economic security dimension includes per capita availability of meat, dairy products, seafood, and fruits, as well as average protein intake—which can indirectly reflect the richness of a region’s food supply—these are per capita supply-side indicators and, due to the unavailability of microdata, were not included. Third, at the conceptual level, there is a distinction between structural security and economic security. The former measures the balance in the allocation of land resources between food crops and cash crops, falling under the level of production input structure; the latter focuses on total final output and per capita supply of physical nutritional inputs, falling under the level of output outcomes. Changes in the food-to-cash crop ratio may affect the output value of cash crops, which in turn affects per capita consumption of cotton, oilseeds, and sugar—a key component of economic security—thus reflecting structural transmission within the agricultural system. If micro-level survey data becomes available in the future, the indicators not included above could be expanded upon.

## 4. Results and Analysis

### 4.1. Analysis of the Overall Level of Supply Security

As shown in Table 4, the average score for food and key agricultural product supply security in Gansu Province rose from 0.2499 to 0.3259, indicating an overall improvement in the region’s supply security capacity; the standard deviation fluctuated upward from 0.0525 to 0.1002. Jinchang, Wuwei, Qingyang, and Pingliang were the leading regions in terms of rapid growth, with cumulative increases ranging from 48.58% to 76.25%, demonstrating the strongest growth momentum. Among them, Jinchang City led the province with a growth rate of 76.25%, while Wuwei City recorded a growth rate of 67.76%, demonstrating the development momentum of the core area of the Hexi Corridor. In eastern Gansu, Pingliang City and Qingyang City achieved growth rates of 51.56% and 48.58%, respectively, highlighting the enormous potential of the dryland agriculture region. Zhangye, Tianshui, Longnan, and Baiyin constitute the steady-growth region, with growth rates ranging from 19.94% to 46.89%, maintaining a stable upward trend. Zhangye achieved robust growth of 46.89% from a relatively high baseline and firmly held the top spot in the province in 2023 with an absolute level of 0.4773. However, the slowdown in growth rates indicates that it is becoming increasingly difficult for some regions with high base figures to sustain high growth levels, and the province’s growth engine urgently needs to shift from relying on individual regions with high base figures to fostering more high-growth regions. Dingxi City, Jiuquan City, Lanzhou City, and Linxia Prefecture are classified as slow-growth areas, with relatively limited growth rates. The cumulative growth rates of these cities and prefectures all fell below 18 percent: Dingxi City grew by 17.21 percent; Jiuquan City, starting from a relatively high base, grew by 16.49 percent; Lanzhou City grew by 12.12 percent; and Linxia Prefecture saw a growth rate of only 6.19 percent. Both their growth bases and rates rank among the lowest in the province, making them key areas requiring attention for balanced regional development. Gannan Prefecture and Jiayuguan City are classified as fluctuating adjustment zones, and their development trajectories have been volatile. Jiayuguan City experienced a peak in 2021 followed by a decline; Gannan Prefecture saw a decline after 2021 but rebounded by 2023 to a level close to that at the beginning of the period, indicating instability in development. This reflects the dynamic adjustment process in ecologically fragile areas and key ecological function zones as they balance conservation and development.

**Figure 3:**
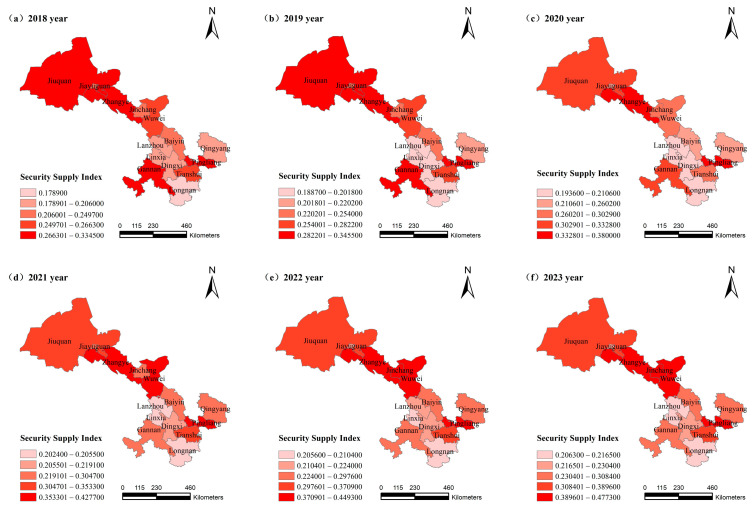
Spatial Distribution Trends of the Comprehensive Index of Food and Key Agricultural Product Supply Security.

The previously mentioned “High-Growth Zone”, “Steady-Growth Zone”, “Slow-Growth Zone”, and “Volatile-Adjustment Zone” are classifications of trends based on cumulative growth rates from 2018 to 2023. The safety levels in Table 4 are classified using natural breakpoints derived from the 2023 composite scores, reflecting the current tier of each prefecture-level city. These two classification dimensions are complementary and help to simultaneously grasp both the current safety status and evolving trends. Combined with ArcGIS 10.8.1 spatial visualization (Figure 3), it can be observed that the security of grain and key agricultural product supplies in Gansu Province exhibits the spatial characteristics of “overall improvement with diverging growth rates”. From a geographic perspective, the high-growth zones are primarily concentrated in the central and eastern parts of the Hexi Corridor and the Longdong region, while the slow-growth zones are mostly located in the Longzhong and Longnan regions.

### 4.2. Robustness Analysis

To avoid the potential randomness in results caused by a single weighting method, the coefficient of variation method was adopted in place of the weighting method to conduct a robustness test. The coefficient of variation method directly uses the ratio of an indicator’s standard deviation to its mean to reflect the degree of variation; it contains no subjective parameters and, together with the entropy-weighted method, forms a complementary pair in terms of information content and dispersion. The entropy weighting method is based on information entropy theory and measures the differences in the amount of information contained in each indicator; in contrast, the coefficient of variation method directly considers the absolute dispersion of the data and focuses on measuring the degree of variation in the observed values [37] (Yang Yu, 2006). In contrast, although the CRITIC method considers both standard deviation and inter-index correlations, it may suffer from the problem of correlations dominating the weights when applied to the high-dimensional set of indicators in this study; the Analytic Hierarchy Process (AHP) relies on expert judgment, which is inconsistent with the objective weighting approach adopted in this study. Therefore, the coefficient of variation method is the most appropriate for conducting robustness tests. The weights of each indicator in the indicator system were recalculated using the coefficient of variation method and reinserted into the TOPSIS model to determine the comprehensive supply security scores for each prefecture and city under the coefficient of variation method.

The raw comprehensive supply security scores derived from the entropy-weighted TOPSIS method and the alternative scores obtained using the coefficient of variation method were, respectively, converted into annual rankings. A consistency test was conducted using Spearman’s rank correlation coefficient. As shown in Table 5, Spearman’s rank correlation coefficients for 2018–2023 were all higher than 0.95, indicating a significant and strong positive correlation. This indicates that the comprehensive scores for the supply security of grain and key agricultural products in Gansu Province, derived using the alternative weighting method, are highly consistent with those of the original approach. This, to a certain extent, mitigates the potential bias in the index measurement that might result from differences in weighting methods.

As shown in Table 6, among the top six cities in Gansu Province’s 2023 comprehensive score for the security of grain and key agricultural product supply—Zhangye, Wuwei, Jinchang, Pingliang, Jiuquan, and Tianshui—only Wuwei, Jinchang, and Pingliang experienced slight shifts in ranking between 2nd and 4th place, while the rankings of the remaining cities did not change by more than two positions. This indicates that, regardless of the weighting method used, the relative rankings of Gansu Province’s food and key agricultural product supply security remain stable, and the overall spatial differentiation patterns have not changed. Consequently, the conclusions—such as Zhangye City firmly holding the top position and the Central and Eastern Hexi Corridor and Longdong regions being high-growth areas—are robust and reliable.

### 4.3. Analysis of Supply Security by Dimension

**Figure 4:**
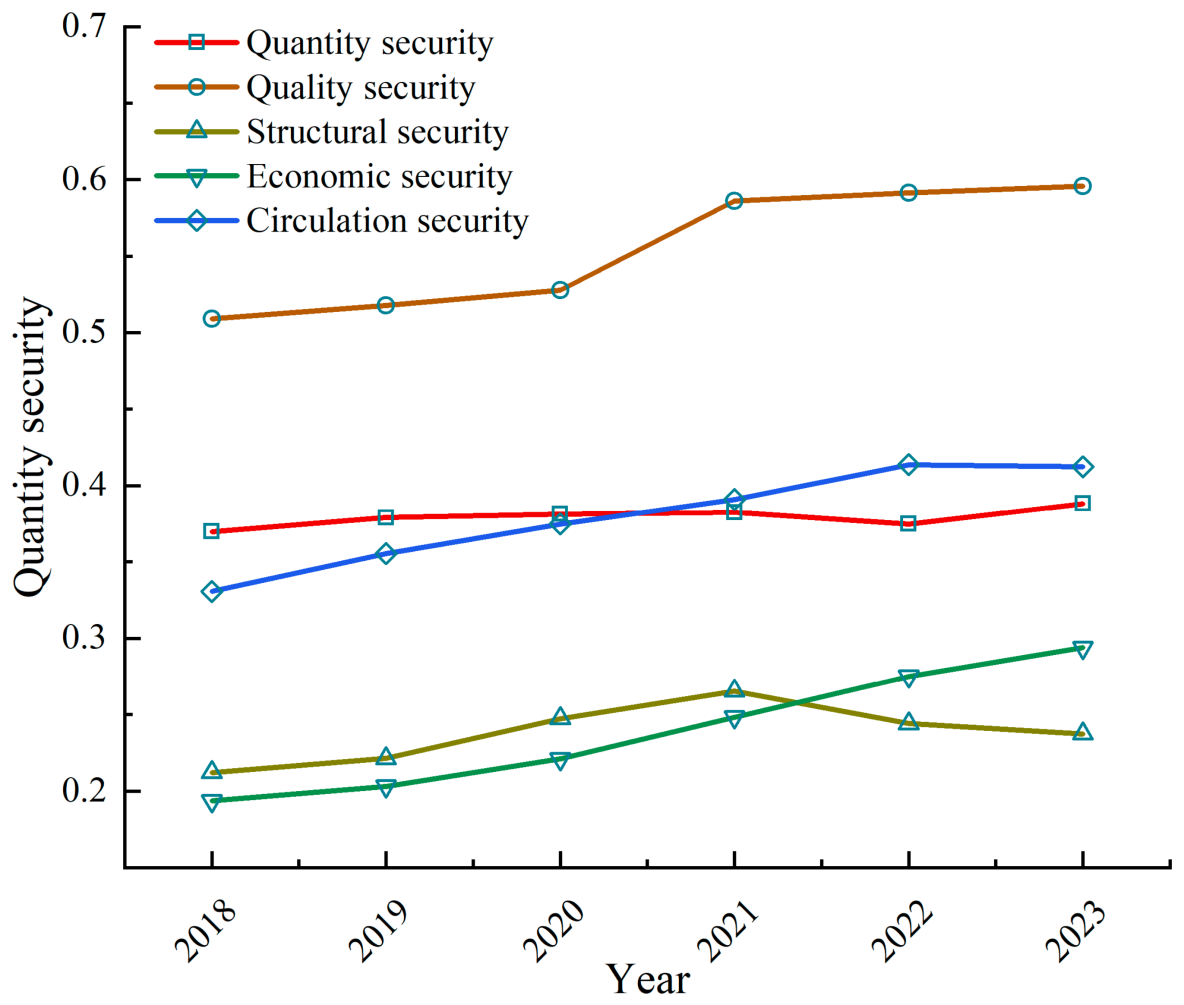
Multi-dimensional Security Supply Index for Grain and Key Agricultural Products.

The trends in the various dimensions of food security for grain and key agricultural products in Gansu Province from 2018 to 2023, viewed through the lens of a comprehensive food security framework, are as follows (Figure 4):

The supply security level fluctuated upward from 2018 to 2023, with the index rising from 0.3698 to 0.3881. During this period, it grew steadily from 2019 to 2021, declined slightly in 2022, and rebounded in 2023, surpassing the previous high. This upward cycle coincided with the implementation of policies such as the development of the “Two Zones” (grain production functional zones and key agricultural product production protection zones), indicating a synergistic trend with the optimization of industrial layout. Following the national policy rollout in 2017, Gansu Province’s implementation measures began to yield concentrated results in 2018 and beyond. By demarcating and developing contiguous production protection zones, the province promoted the construction of high-standard farmland, the installation of efficient water-saving irrigation facilities, and the large-scale adoption of high-quality seeds and advanced farming techniques, thereby directly strengthening the foundation of agricultural production capacity. The temporary decline in 2022 may have been related to natural disasters in certain regions, reflecting that the production capacity base remains vulnerable to disruptions from natural risks. The rapid rebound in 2023, however, indicates that the infrastructure and production capacity advantages accumulated in previous periods possess strong resilience.

The quality and safety of supply have improved significantly, with the index rising steadily from 0.5092 in 2018 to 0.5958 in 2023, making it the dimension with the highest safety level. This has progressed in tandem with the in-depth implementation of major strategies such as ecological conservation and high-quality development in the Yellow River Basin. Compared to the early stages of the 13th Five-Year Plan, there have been continuous improvements during the period in areas such as water-saving irrigation and soil and water conservation efforts, and the initiative to reduce the use of pesticides and chemical fertilizers while enhancing their efficiency has been vigorously advanced, resulting in a significant enhancement of the environmental carrying capacity and sustainability of agricultural production.

The level of security in the supply structure fluctuated significantly, with the index ranging from 0.2123 to 0.2655, reflecting the process of the industrial structure seeking equilibrium amid dynamic adjustments. This was primarily due to the ongoing impact of the coordinated development strategy for “grain, cash crops, and fodder”, as well as dynamic adjustments in the production relationship between high-yield crops such as corn and potatoes and specialty cash crops such as traditional Chinese medicinal materials and highland summer vegetables. At the same time, the focused approach of the “Two Zones” initiative has led to greater spatial concentration and clearer functional roles in grain production, thereby fostering a more distinct regional layout and complementary mechanisms with specialty industries.

The Economic Security Index for Food Supply rose from 0.1938 to 0.2940, reflecting an overall improvement in agricultural productivity and residents’ ability to access food. Since 2018, a provincial-level agricultural insurance risk protection network has been established. By 2023, the number of insurance products had reached 101, covering all specialty industries such as cattle, sheep, vegetables, fruits, potatoes, and medicinal herbs. By mitigating natural and market risks, this network has ensured the stability of per capita production of meat, dairy products, fruits, and other commodities; At the same time, the grain industry economy has been vigorously developed, with efforts to advance processing and value-added transformation as well as the development of the entire industrial chain. This has directly driven growth in the total output value of agriculture, forestry, animal husbandry, and fisheries, while also boosting employment and increasing incomes.

The Supply Chain Security Index rose steadily from 0.3307 to 0.4123, indicating sustained progress in the development of warehousing and logistics facilities, transportation networks, and market systems. In 2017, the “Implementation Opinions on Further Promoting Logistics Cost Reduction and Efficiency Improvement to Foster the Development of the Real Economy” was implemented, introducing measures such as improving urban distribution and establishing national-level logistics hubs. Subsequent plans emphasized strengthening the construction of cold-chain logistics facilities for the storage and preservation of agricultural products, as well as optimizing the logistics industry layout of “one center, four hubs, and five nodes”, providing systematic support for enhancing distribution efficiency.

### 4.4. Analysis of Factors Affecting the Level of Supply Security

Based on the above analysis, we examine the factors affecting the supply security of grain and key agricultural products in Gansu Province. Drawing on the research by Zhang Jiawei et al. (2026) [36] and Han Qunzhu et al. (2020) [38] and taking into account the potential impact of regional economic development, irrigation infrastructure, and agricultural labor input on supply security, we have established the following indicators (Table 7). Among these, per capita regional gross domestic product (GDP), as a core indicator of regional economic strength, reflects the capacity of the economic foundation to support agricultural infrastructure development and technological innovation; the employment rate in agriculture, animal husbandry, fisheries, and forestry measures the scale of labor input and serves as a fundamental condition for ensuring the smooth operation of agricultural production; The proportion of effectively irrigated area is a key indicator of a region’s capacity to ensure water security and the level of agricultural water conservancy infrastructure; the urbanization rate reflects the transformation of a region’s urban–rural structure; and the crop disaster rate measures the severity of the impact of natural disasters on agricultural production.

As shown in Table 8, the overall significance test statistic for the least squares model is F = 5.704, with *p* = 0.000, indicating that the model as a whole is statistically significant at the 1% significance level; that is, the factor variables in the model collectively have a significant effect on the dependent variable (the overall level of food and key agricultural product supply security). Regarding the specific impact of each variable, the proportion of effectively irrigated area is a positive driver of supply security (β = 0.001, *p* < 0.01). This variable passed the 1% significance test, indicating that for every 1 percentage point improvement in irrigation conditions, the comprehensive supply security score increases by an average of approximately 0.001 units. Gansu Province is an arid and semi-arid region, where water is the lifeblood of agriculture; the larger the area under effective irrigation in a city or prefecture, the higher its level of supply security. Per capita regional GDP has a significant positive impact on supply security (β = 0.020, *p* < 0.01). For every 10,000 yuan increase in per capita GDP, the supply security score rises by approximately 0.020 units. This further validates, at the prefecture-level city and autonomous prefecture level, the logic that economic foundations determine agricultural investment capacity: regions with stronger economic strength are better able to advance agricultural infrastructure development, agricultural technology extension, and risk mitigation systems. The crop damage rate is a negative factor affecting supply security (β = −0.004, *p* < 0.01); as it passed the 1% significance test, this indicates that the role of disaster prevention and mitigation capabilities in ensuring supply security is even more urgent. The employment rates in agriculture, animal husbandry, fisheries, and forestry, as well as the urbanization rate, did not pass the significance test. The lack of a significant employment rate in agriculture, animal husbandry, fisheries, and forestry may be related to the continued outflow of agricultural labor and the ongoing increase in agricultural mechanization in Gansu Province during the sample period, indicating that agricultural production methods are shifting from labor-intensive to capital- and technology-intensive models. The fact that the urbanization rate has not risen significantly indicates that the displacement of rural labor by urbanization has not yet had a negative impact on the security of the labor supply.

## 5. Research Discussion

### 5.1. Spatial Differentiation

Cities such as Zhangye and Wuwei, which scored highly in Gansu Province’s assessment of food and key agricultural product supply security, are precisely the province’s main commercial grain production bases. The central and eastern parts of the Hexi Corridor have effectively boosted agricultural productivity thanks to favorable irrigation conditions [39]. Coupled with the high coverage rate of high-standard farmland and the significant clustering effects of specialty industries such as seed corn production [40], these areas have effectively capitalized on policy and investment dividends; however, they also harbor risks of land diversion from grain production and resource pressures [12]. The pattern of faster progress in the west and slower progress in the east, along with diverging growth rates, indicates an uneven implementation of the strategy, consistent with the conclusion reached by Wang Huogen et al. [17] and Jiang et al. [18] that improvements in safety levels are typically accompanied by internal disparities. Land consolidation can significantly reduce rural vulnerability by enhancing natural and physical capital, and this effect exhibits spatial variation depending on distance from water sources [41]. However, in areas such as Central Longshan, the scale economies of mechanized operations are difficult to realize due to ecological protection red lines and topographical constraints. Furthermore, the combination of fragmented farmland and water constraints further exacerbates the loss of returns on infrastructure investment, This phenomenon echoes the findings of Andrianarimanana et al. (2023) in their empirical study of Madagascar, where a tropical climate, deteriorating soil quality, and infrastructure deficiencies have undermined the country’s agricultural productivity and the international comparative advantage of its agricultural products [42], revealing that the combined effect of natural resource constraints and infrastructure deficiencies on agricultural efficiency is a common cross-regional phenomenon. Zhong Yu and Gan Linzhen (2022) [43], in their study on food security in the arid regions of Northwest China, similarly demonstrated that water scarcity is the core bottleneck constraining grain production in the arid areas of Gansu and Shaanxi, and that the steadily increasing proportion of drought-tolerant crops—such as corn and potatoes—is also a key structural factor driving grain yield increases in these arid regions; Ma Yuan et al. (2024) [44], focusing on water scarcity and food security risks in Central Asia, pointed out that the efficiency of water resource allocation is a key variable determining the resilience of agricultural systems in arid regions, confirming that irrigation conditions and crop structure adaptation jointly drive food security in arid regions.

From all perspectives, the highest level of quality and safety, with significant improvements, aligns with the interpretation of the strategic implications of the “comprehensive food security” approach by Cheng Guoqiang et al. [8] and is consistent with the assessment by Gao Ming and Zhao Xue [6] and Zhou Guohua et al. [5] Regarding the need for multidimensional, coordinated efforts to strengthen the foundation of food security, this is because policies to reduce pesticide and fertilizer use have an immediate emission-reduction effect; the dual-reduction targets are clear and subject to strict performance evaluations, and negative indicators can be significantly improved in the short term through the promotion of agricultural technologies [45]. Qiao et al. (2022) [46] examined the importance of soil quality in agricultural production; however, in this study, the quality and safety dimension was characterized solely by the use of chemical fertilizers and pesticides, failing to include quality metrics such as soil quality and the pass rate of pesticide residue spot checks, which may have led to a slight overestimation of the degree of improvement in this dimension. In contrast, quantitative security is constrained by the fixed limits on arable land and the increasing marginal costs of raising yields [47], resulting in relatively linear growth. Structural security involves adjustments to crop patterns and the restructuring of industrial chains. The case of Switzerland’s livestock industry demonstrates that while a shift in crop patterns from feed crops to legumes holds environmental and health benefits, it requires comprehensive adjustments in policy, pricing, and breeding, and cannot be achieved overnight [48]. Béné et al. (2019) also noted in their analysis of the sustainable transformation of global food systems that improvements in environmental performance often precede changes in dietary patterns [49]. Renard and Tilman (2019), based on a large-scale study spanning 50 years across 91 countries and 176 crop species, found that the higher the crop diversity at the national level, the greater the temporal stability of total national production [50]. The fluctuations in the structural security dimension in Gansu, a major livestock-producing province, echo the issue of mismatch between grain and forage structures highlighted by Liu Changquan et al. [25] and serve as a regional cautionary note regarding the nutrition-oriented, demand-driven production transition advocated by Liang Yajia and Chen Kunqiu [13].

### 5.2. International Comparison and Reflection on Development Pathways

The Food and Agriculture Organization of the United Nations (FAO) proposed the four pillars of food security—availability, accessibility, utilization, and stability—with a focus on end-consumer needs and the protection of individual well-being. Li et al. (2026) conducted a bibliometric analysis of the global food security literature from 1990 to 2022, revealing that the concept of food security has evolved from the traditional three-pillar and four-pillar frameworks to a six-dimensional framework that incorporates agency and sustainability, reflecting the global academic community’s focus on equity in food systems and intergenerational balance [51]. This study constructs a five-dimensional framework comprising “quantity, quality, structure, economy, and distribution”, placing greater emphasis on identifying internal structural shortcomings in regional food security from the perspectives of production and distribution. It represents a supply-side extension of the classic FAO framework at the provincial and prefectural levels. In particular, the inclusion of structural security and distribution security as independent dimensions goes beyond the traditional evaluation paradigm centered on production volume or macro-level supply–demand fluctuations, such as the assessment of food security in South Asian countries conducted by Kousar et al. [19]. However, compared to the study by Li Shaokun et al. (2026) [52], which identified global trends in food security improvements based on indicators such as the cost and affordability of healthy diets, this study—due to limitations in the availability of microdata at the prefecture and city levels—was unable to incorporate dimensions such as affordability and dietary diversity; consequently, its evaluation results are more applicable to identifying structural shortcomings at the regional production level. Long Wenjin et al. (2025) [53] examined the transformation of China’s food system since 2013. The study period covered 2018–2023, a relatively short timeframe. While this period captures trends in food security levels in recent years, it is difficult to identify cyclical fluctuations and structural changes over longer time scales; further research involving longer-term tracking is needed.

Regarding development pathways, international scholar Timmer (2012) points out that the psychological impact of food price volatility far exceeds the numerical values themselves; since price stability is a public good that the market cannot provide, the government therefore bears the responsibility for intervening to prevent food crises [54]. Unlike international research, which focuses on the impact of market volatility and climate shocks on household-level resilience, the Gansu case demonstrates that national planning and infrastructure investment have a significant impact on regional supply capacity. This approach aligns with trends such as the emphasis on science and technology innovation as a driver highlighted by Wang Xiaojun and Mao Shiping [28] and the development of supply chain resilience studied by Yang et al. [22], all of which aim to enhance the overall competitiveness of agriculture through systematic investment. Li Shaokun et al. (2026) [51] point out that, due to the combined effects of agricultural pollution, food waste, supply chain disruptions, and climate change, global food security trends have taken a worrying turn since 2015, and that climate shocks and price volatility have exacerbated access difficulties in low-income regions. The pattern of uneven growth within Gansu Province also indicates that, even with strong policy and investment interventions, local agricultural and land conditions remain the determining factors in regional resilience. This insight is fundamentally consistent with the conclusions reached by Siderius et al. [15] regarding the impact of water resource management on food self-sufficiency rates, and it aligns with the fundamental challenges faced by many resource-constrained regions around the world.

## 6. Conclusions and Recommendations

### 6.1. Research Findings

Based on a “broad food perspective”, this study conducts a dynamic assessment of food security levels for grain and key agricultural products across 14 prefectures and cities in Gansu Province from 2018 to 2023. The study found that Gansu Province’s food security levels have steadily improved, showing an overall upward trend with varying growth rates across regions. Growth has been robust in the central and eastern parts of the Hexi Corridor and in eastern Gansu, while growth rates in some areas of central and southern Gansu have lagged. In terms of dimensions, quality and safety have reached the highest level and shown significant improvement; economic safety has seen the largest increase and serves as the primary driving force; quantitative safety has strengthened amid fluctuations; distribution safety has generally improved steadily; and structural safety has fluctuated and been optimized through the coordinated management of grain, cash crops, and fodder. An analysis of influencing factors further indicates that per capita regional GDP and the proportion of effectively irrigated land are the primary positive drivers of improved supply security, while the crop disaster rate is the main constraining factor. The scientific contribution of this study lies in the development of a multidimensional evaluation framework that better aligns with the essence of the “comprehensive food perspective”. By integrating the Global Food Security Index with the HLPE’s sustainability principles and linking the input structure with output outcomes, the framework incorporates per capita consumption of meat, dairy, fruits, and other food items, as well as average protein supply, thereby addressing the inherent requirement for the “comprehensive food perspective” to transition toward nutrition and health; It reveals the spatiotemporal patterns of supply security in Gansu Province—a region in Northwest China characterized by a balance between production and consumption—and provides a scientific basis for formulating differentiated agricultural support policies. Future research could extend the time span, incorporate demand-side indicators, and conduct causal analysis and cross-provincial validation to promote integrated assessment of both supply and demand sides and the continuous refinement of the framework.

### 6.2. Recommendations for Countermeasures

Taking into account the divergent characteristics of regional development, this study proposes a differentiated and coordinated regulatory approach: regions experiencing slower growth should prioritise addressing infrastructure shortcomings, while regions with comparative advantages should strengthen industrial integration and technological empowerment; in ecologically fragile areas, water-saving incentives and measures to improve the quality of arable land should be implemented; and, in parallel, the comprehensive institutional framework—including ecological compensation, the expansion of insurance coverage and the regulation of agricultural inputs—should be refined across the board. Specifically:

(1) Implement targeted regional empowerment by selecting 3–5 counties or districts annually in the central and eastern parts of the Hexi Corridor and the Longdong region to establish demonstration parks for integrated grain industry development, complete with supporting infrastructure and the targeted deployment of science and technology specialists; in the Central Longdong and Longnan regions, incorporate the rehabilitation of low- and medium-yield farmland into performance assessments, establish pre-cooling facilities at production sites, and promote the cultivation of local brands and the extension of ‘Science and Technology Villages’ into specialised production areas.
(2) We will consolidate the foundations of supply security by carrying out comprehensive land consolidation in suitable areas, improving the quality of arable land, optimising its distribution, and strictly enforcing quality inspections and subsequent maintenance and management in relation to the ‘land-for-land’ compensation scheme.
(3) Promote multi-dimensional security coordination; establish a provincial-level ecological compensation mechanism; introduce a water-saving incentive scheme in drought-prone areas, linking compensation to the effectiveness of water-saving irrigation and soil and water conservation measures; at the same time, support leading enterprises in achieving industrial integration and provide them with financial support, whilst expanding the coverage of full-cost insurance and income insurance.
(4) Promote green and sustainable production models, strengthen market supervision and accountability in the agricultural inputs sector, tap into reserve arable land resources, and consolidate the foundations of food security from multiple angles.

### 6.3. Research, Innovation, and Future Prospects

The contribution of this study lies in the fact that, given Gansu Province’s status as one of the nation’s 11 provinces with a balanced grain production and consumption system, it incorporates distinctive and competitive agricultural products, such as potatoes, beef, mutton, and highland summer vegetables, as well as nutritional indicators like protein supply into the evaluation system. The study has established an evaluation index system for the secure supply of grain and key agricultural products tailored to Gansu’s specific characteristics, revealing the spatiotemporal patterns of intra-regional food security. This provides a basis for formulating differentiated agricultural support policies that align with local conditions. However, the following limitations remain. First, the study relies on official secondary statistical data and fails to incorporate micro-level household survey data to reflect actual household consumption patterns. Although the selection of indicators is based on a theoretical framework and the literature, the categorization of dimensions is somewhat subjective. The study does not include indicators for affordability and dietary diversity, and the number of indicators under the “structural safety” dimension is relatively small, which may result in an insufficient description of the impact on crop structure. Future research could incorporate indicators such as the proportion of forage and feed to improve the analysis. Second, the study period (2018–2023) is relatively short, making it difficult to capture long-term trends. The conclusions are based on the agricultural resources and industrial characteristics of Gansu Province. In the future, the time span could be expanded, and cross-regional comparisons could be conducted with other provinces that maintain a balance between grain production and consumption. Indicators could also be adjusted based on the distinctive crops of other regions to enhance the applicability of the “comprehensive food perspective” evaluation framework developed in this study across a broader geographical area. Third, the entropy-weighted method assumes that the various indicators are independent of one another, but some indicators may be correlated. Robustness tests have been conducted using the coefficient of variation method, which has mitigated this issue to some extent. Additionally, the entropy-weighted TOPSIS model cannot directly establish a causal relationship between policies—such as the “Two Zones” planning and the Yellow River Basin Strategy—and the security index. In the future, econometric methods such as the double-difference method could be employed to identify causal relationships. Fourth, regarding external validation, there is a lack of independent quantitative benchmarks for food security at the sub-provincial level. If independent data on food consumption and nutrition become available in the future, rigorous external criterion-based validation can be conducted.

## Figures and Tables

**Table 1:** Rural Workforce and Per Capita Agricultural Product Availability.

Item\Year	Population (10,000 Persons)	Rural Workforce (10,000 Persons)	Per Capita Grain Consumption (kg)
2018	2515.08	789	590.99
2019	2509.02	768	466.82
2020	2501.02	713	532.86
2021	2490.02	693	556.19
2022	2492.42	689	570.84
2023	2465.48	681	580.54

**Table 2:** Gansu Province Specialty Industry Production Analysis Table (Unit: 10,000 tons).

Item\Year	Beef	Mutton	Vegetables	Ornamental Fruits	Potatoes	Cordyceps and Medicinal Herbs
2018	21.40	23.60	1292.57	370.03	202.33	101.85
2019	22.74	25.00	1388.75	438.54	206.85	113.15
2020	24.90	27.60	1478.51	481.07	222.82	123.22
2021	27.00	33.50	1655.25	539.07	224.61	131.46
2022	27.18	36.52	1736.64	575.39	222.64	137.48
2023	29.76	40.90	1822.65	616.40	222.10	148.90

**Table 3:** Comprehensive Evaluation Index System for Food and Key Agricultural Product Supply Security.

Dimension	Indicator	Indicator Attribute
Quantity Security	Per capita arable land area	Positive
	Rural workforce	Positive
	Agricultural labor force education level	Positive
	Total Agricultural Machinery Power	Positive
	Contribution rate of agricultural technological progress	Positive
Quality and Safety	Fertilizer Application Volume	Negative
	Pesticide Usage	Negative
Structural Safety	Grain-to-cash ratio	Positive
	Grain-oil ratio	Positive
Economic Security	Total Output Value of Agriculture, Forestry, Animal Husbandry, and Fisheries	Positive
	Per capita grain availability	Positive
	Per capita cotton, oil, and sugar availability	Positive
	Per capita milk consumption	Positive
	Per capita egg and poultry consumption	Positive
	Per capita meat consumption	Positive
	Per capita seafood consumption	Positive
	Per capita fruit consumption	Positive
	Average protein supply	Positive
Distribution Safety	Transportation route density	Positive
	Highway freight mileage	Positive

**Table 4:** Comprehensive Score Index for Food and Key Agricultural Product Supply Security.

Item	2018	2019	2020	2021	2022	2023	Cumulative Growth Rate		2023 Score Categories
							Growth Rate	Ranking	
Lanzhou City	0.1906	0.2018	0.2005	0.2044	0.2169	0.2137	12.12%	11	Low
Jiayuguan City	0.2382	0.2529	0.2508	0.2824	0.2224	0.2275	−4.48%	14	Lower
Jinchang City	0.2497	0.2498	0.2834	0.3016	0.4005	0.4401	76.25%	1	High
Baiyin City	0.2466	0.2540	0.2602	0.2753	0.2864	0.2958	19.94%	8	Medium
Tianshui City	0.2663	0.2822	0.2992	0.3232	0.3345	0.3525	32.36%	6	Higher
Wuwei City	0.2627	0.2672	0.3029	0.3811	0.4088	0.4407	67.76%	2	High
Zhangye City	0.3250	0.3324	0.3541	0.4277	0.4493	0.4773	46.89%	5	High
Pingliang City	0.3022	0.3455	0.3800	0.4136	0.4353	0.4580	51.56%	3	High
Jiuquan City	0.3345	0.3376	0.3294	0.3533	0.3709	0.3896	16.49%	10	Higher
Qingyang City	0.2060	0.2202	0.2413	0.2780	0.2976	0.3060	48.58%	4	Medium
Dingxi City	0.1966	0.1959	0.2106	0.2191	0.2240	0.2304	17.21%	9	Lower
Longnan City	0.1789	0.1887	0.1936	0.2024	0.2104	0.2165	20.99%	7	Low
Linxia Hui Autonomous Prefecture	0.1943	0.2012	0.2041	0.2055	0.2056	0.2063	6.19%	12	Low
Gannan Tibetan Autonomous Prefecture	0.3078	0.3146	0.3328	0.3047	0.2972	0.3084	0.21%	13	Medium
Mean	0.2499	0.2603	0.2745	0.2980	0.3114	0.3259	-	-	-
Standard Deviation	0.0525	0.0554	0.0610	0.0758	0.0889	0.1002	-	-	-

**Table 5:** Test of the Rank Correlation Coefficient Between the Results of the Entropy-Weighted Method and the Coefficient of Variation Method.

Year	Spearman’s Rank Correlation Coefficient
2018	0.9736
2019	0.9604
2020	0.9736
2021	0.9516
2022	0.9692
2023	0.9516

**Table 6:** 2023 Composite Scores and Rankings.

Item	Entropy-Weighted Method		Coefficient of Variation Method	
	Comprehensive Supply Security Score	Ranking	Comprehensive Supply Security Score	Ranking
Lanzhou City	0.2137	13	0.2546	14
Jiayuguan City	0.2275	11	0.2598	13
Jinchang City	0.4401	4	0.4456	3
Baiyin City	0.2958	9	0.3466	8
Tianshui City	0.3525	6	0.3628	6
Wuwei City	0.4407	3	0.4704	2
Zhangye City	0.4773	1	0.5030	1
Pingliang City	0.4580	2	0.4398	4
Jiuquan City	0.3896	5	0.4295	5
Qingyang City	0.3060	8	0.3518	7
Dingxi City	0.2304	10	0.2868	10
Longnan City	0.2165	12	0.2687	11
Linxia Hui Autonomous Prefecture	0.2063	14	0.2604	12
Gannan Tibetan Autonomous Prefecture	0.3084	7	0.3241	9

**Table 7:** Factors Affecting the Level of Food and Key Agricultural Product Supply Security in Gansu Province.

Dependent Variable	Independent Variable	Indicator Description
Overall Level of Security in the Supply of Grain and Key Agricultural Products	Per Capita Regional Gross Domestic Product	Regional Gross Domestic Product/Permanent Resident Population
	Employment Rate in Agriculture, Forestry, Animal Husbandry, and Fisheries	Number of Workers in Agriculture, Forestry, Animal Husbandry, and Fisheries/Total Employment
	Proportion of Land Under Effective Irrigation	Area Under Effective Irrigation/Total Cropland Area
	Urbanization Rate	Urban Permanent Resident Population/Total Permanent Resident Population
	Crop Damage Rate	Area Affected by Disasters/Total Cropland Area

**Table 8:** Results of the Linear Regression (Least Squares) Model.

Explanatory Variables	Unstandardized Coefficient	Standard Error	Standardized Coefficient	t-Value	*p*-Value
Constants	0.342	0.090	-	3.800	0.000 ***
Per Capita Regional GDP	0.020	0.007	0.794	2.849	0.006 ***
Employment Rate in Agriculture, Animal Husbandry, Fisheries, and Forestry	0.000	0.001	0.019	0.194	0.846
Proportion of Effectively Irrigated Area	0.001	0000	0.381	3.099	0.003 ***
Urbanization Rate	0.001	0.001	0.088	0.878	0.383
Crop Damage Rate	−0.004	0.001	−0.891	−3.402	0.001 ***

Note: *** represent significance levels of 1%, respectively.

## Data Availability

The original contributions presented in this study are included in the article/Supplementary Materials. Further inquiries can be directed to the corresponding authors.

## Supplementary Materials

The following supporting information can be downloaded at: https://www.mdpi.com/article/10.3390/foods15152687/s1.

## References

[1] Song X., Zhang C.P., Zou X.M., Zhang Y. (2024). To ensure China’s stable and safe supply of grain and important agricultural products. Macroecon. Manag..

[2] Zhao L. (2024). The Current Situation and Strategic Planning of Ensuring the Supply Security of Primary Products in China. Pac. J..

[3] Wang H.R. (2022). Pursuing a Path to Food Security with Chinese Characteristics. Chin. Econ. Wkly..

[4] Xu X.P., Yin Q., Zhang F., Chen Y.G. (2025). Modernization of agricultural and rural areas and comprehensive rural revitalization for the “15th Five-Year Plan” period: Core issues and implementation pathways. J. Agric. For. Econ. Manag..

[5] Zhou G.H., Long H.L., Lin W.L., Qiao J.J., Tan H.Y., Yang K.Z., Yue W.Z., Yun W.J., Huang X.J., Lu H.W. (2023). Theoretical debates and practical development of the “three rural issues” and rural revitalization in the New Era. J. Nat. Resour..

[6] Gao M., Zhao X. (2023). Grain security from the perspective of agricultural power: Realistic foundation, problem challenges and promotion strategies. Res. Agric. Mod..

[7] Qiu H.G., Lei X.Y., Leng G.S., Liu M.Y. (2022). A Comprehensive Theoretical Analysis of Grain Security in the New Era. Chin. Rural Econ..

[8] Cheng G.Q. (2023). The Greater Food Approach: Structural Change, Policy Implications and Practice Logic. Issues Agric. Econ..

[9] Li Y.H., Huang Y. (2025). A Study on the Challenges Facing Food Security in the Three Northeastern Provinces Under a Holistic Approach to Food and Solutions to These Challenges. Agric. Econ..

[10] Song M., Zhang A.L. (2023). Cultivated Land Use Transition from the “Greater Food” Perspective: Realistic Challenges, Theoretical Logic and Implementation Paths. China Land Sci..

[11] Huang D., Lu Y., Tong Z., Pang B., Liu Y., Liu Y. (2026). Governing non-grain production of farmland: A differentiated strategy based on grain production loss risk. Land Use Policy.

[12] Fan S.G., Tian X., Long W.J. (2024). Challenges and Countermeasures of Food Supply-Demand Balance in China Under the Greater Food Approach. J. Huazhong Agric. Univ. (Soc. Sci. Ed.).

[13] Liang Y.J., Chen K.Q. (2025). Nutrient supply and demand evolution of China’s agri-food system under greater food approach. Resour. Sci..

[14] Ofoedu E.C., Bozkurt H., Mortimer C.J. (2025). Towards sustainable food security: Exploring the potential of duckweed (Lemnaceae) in diversifying food systems. Trends Food Sci. Technol..

[15] Siderius C., Velde D.V.Y., Gülpen M., de Bruin S., Biemans H. (2024). Improved water management can increase food self-sufficiency in urban foodsheds of Sub-Saharan Africa. Glob. Food Secur..

[16] Vidanagamachchi K., Ginige A., Nakandala D. (2025). Viable agri-food supply chains: Survival through systemic adaptations. Systems.

[17] Wang H.G., Hu M.T., Liu X.C. (2025). Reconstruction and interpretability analysis of China’s food security level based on machine learning and SHAP algorithm. J. China Agric. Univ..

[18] Jiang Q., Rong Z., Yuan Z. (2023). Research on the construction of China’s provincial food security evaluation system and regional performance—Based on the “great food view”. Agriculture.

[19] Kousar S., Ameer A., Nasir A., Afzal M., Abbas S. (2024). Comparative analysis to assess food security in South Asian nations: An application of multivariate GARCH model. Environ. Dev. Sustain..

[20] Pu B.Z., Wei G.C., Li T.Y. (2025). The Application Prospects of Machine Learning in Agricultural Economics Research: Taking Grain as an Example. Issues Agric. Econ..

[21] Yang C., Lu X.H., Xu J.L. (2024). Evaluation and Dynamic Evolution of Food Security Level Based on Big Food Concept. Econ. Geogr..

[22] Yang Y., Qi C., Gu Y., Gui C. (2025). Coordinated development and spatiotemporal evolution trends of China’s agricultural trade and production from the perspective of food security. Foods.

[23] Zhang Y.M., Long W.J. (2023). Challenges and Countermeasures for Improving the Resilience of Agricultural Industry Chain Under the Great Food Outlook. J. Zhongzhou Univ..

[24] Yan T.W., Huang Y.Z. (2025). Theoretical Connotations and Practical Pathways of Agri-food System Transformation under the Big Food Concept—A Case Study of the Edible Mushroom Industry. World Agric..

[25] Liu C.Q., Han L., Li T.T., Wang S.K., Luo Q.F. (2023). The Security of Feed Grains Supply in China from the Perspective of a Big Food Concept. Chin. Rural Econ..

[26] Zhang L.M. (2020). An investigation into the implementation status of the “grain-to-feed” policy: A case study of Gansu. Gansu Agric..

[27] Huang J.K. (2024). New Quality Productive Forces in Agriculture: Connotation and Denotation, Potential and Challenges, and Development Ideas. China Rural Surv..

[28] Wang X.J., Mao S.P. (2023). The achievements of China’s agricultural science and technology innovation and development as socialism with Chinese characteristics for a new era. Sci. Technol. Rev..

[29] Pan D.D. (2025). An Analysis of the Role of Agricultural Insurance in Safeguarding China’s Food Security. Agric. Econ..

[30] Odunitan-Wayas F.A., Faber M., Mendham A.E., Goedecke J.H., Micklesfield L.K., Brooks N.E., Christensen D.L., Gallagher I.J., Myburgh K.H., Hunter A.M. (2021). Food security, dietary intake, and foodways of urban low-income older South African women: An exploratory study. Int. J. Environ. Res. Public Health.

[31] Adong A., Kornher L., Chichaibelu B.B., Amondo E.I., Bashaasha B. (2026). Using high-frequency data to measure household resilience to food insecurity and women’s dietary diversity in Uganda. Food Secur..

[32] Elkenawy S., Alhussan A.A., Gaber K.S., Mattar E.A., Eid M.M., Zaman S., Saber M. (2026). Hybrid metaheuristic feature selection with binary puma optimizer–stochastic fractal search for robust potato price prediction. Potato Res..

[33] Xie H., Wang B. (2017). An empirical analysis of the impact of agricultural product price fluctuations on China’s grain yield. Sustainability.

[34] Zhang Y., Jiang Y.J. (2021). An Entropy-Weighted TOPSIS-Based Performance Evaluation of Agricultural Ecological Environment Governance in Henan Province. South. Agric..

[35] Yang Y.Y., Qi C.J., Dong Y.N., Gu Y.M. (2025). Comprehensive evaluation of China’s food security under the greater food approach: Based on entropy TOPSIS model. J. China Agric. Univ..

[36] Zhang J.W., Pei T.T., Chen Y., Xie B.P. (2026). Assessment of grain and important agricultural products supply security and influencing factors in Gansu Province under the macro-food concept. Arid. Land Geogr..

[37] Yang Y. (2006). An Analysis of Weighting Methods in Multi-Criteria Evaluation. Stat. Decis..

[38] Han Q.Z., Feng Q., Gao H.D., Chen G.P. (2020). Influencing factors of agricultural production changes in Guanzhong based on PCA. Arid. Land Geogr..

[39] Li B., Zhang F., Feng Q., Wei Y., Li G., Song Z. (2025). Research on Grain Production Services in the Hexi Corridor Based on the Link Relationship of “Water–Soil–Carbon–Grain”. Land.

[40] Deng H., Pan X., Zhang H., Xiao Z., Xiao R., Zhao Z., Chen T. (2023). Comprehensive Regulation of Water–Nitrogen Coupling in Hybrid Seed Maize in the Hexi Oasis Irrigation Area Based on the Synergy of Multiple Indicators. Water.

[41] Wei Z. (2021). The impact of land consolidation on vulnerability of rural households: Evidence from Central China. J. Environ. Plan. Manag..

[42] Harinaivo M.A., Pu Y., Finaritra M.T.R. (2023). Assessment of the importance of climate, land, and soil on the global supply of agricultural products and global food security: Evidence from Madagascar. Food Policy.

[43] Zhong Y., Gan L.Z. (2022). Study on the Path of Ensuring Grain Security in Northwest Arid Area Under the Constraint of Resources. Acad. J. Zhongzhou.

[44] Ma Y., Li Y.P., Huang G.H., Liu Y.R., Zhang Y.F. (2024). Collaborative management of water-energy-food-ecosystems nexus in Central Asia under uncertainty. Water Resour. Res..

[45] Hu D., Lu Q., Huang H., Yu H., Xie B., Dong B. (2026). Does Ecological Compensation Reform Enhance the Efficiency of Agricultural Eco-Product Value Realization? Evidence from China. Sustainability.

[46] Qiao L., Wang X.H., Smith P., Fan J.L., Lu Y.L., Emmett B., Li R., Dorling S., Chen H.Q., Liu S.G. (2022). Soil quality both increases crop production and improves resilience to climate change. Nat. Clim. Chang..

[47] Winkler K., Fuchs R., Rounsevell M., Herold M. (2025). Six decades of global crop yield increase and cropland expansion from 1960 to 2020. Environ. Res. Commun..

[48] Keller B., Oppliger C., Chassot M., Ammann J., Hund A., Walter A. (2024). Swiss agriculture can become more sustainable and self-sufficient by shifting from forage to grain legume production. Commun. Earth Environ..

[49] Béné C., Oosterveer P., Lamotte L., Brouwer I.D., de Haan S., Prager S.D., Talsma E.F., Khoury C.K. (2019). When food systems meet sustainability—Current narratives and implications for actions. World Dev..

[50] Renard D., Tilman D. (2019). National food production stabilized by crop diversity. Nature.

[51] Li S., Xu J., Wu H., Han J., Zhuang H., Zhang Z., Wu X., Zhang Z. (2026). Paradigm shifts in achieving global food security: Toward a resilient and sustainable future. Food Secur..

[52] Li S.K., Xu J.L., Wu H.Q., Han J.C., Zhuang H.M., Zhang Z.L., Wu X.Y., Zhang Z. (2026). Global food security under a sustainability perspective: Assessment, challenges, and strategies. Geogr. Sustain..

[53] Long W.J., Meng T., Tian X., Fan S.G. (2025). China’s food security and food system governance: Recent developments and global implications. Food Policy.

[54] Peter C.T. (2012). Behavioral dimensions of food security. Proc. Natl. Acad. Sci. USA.

